# Congential scoliosis in Wilson’s disease: case report and review of the literature

**DOI:** 10.1186/1471-2482-14-71

**Published:** 2014-09-24

**Authors:** Zheng Li, Xin Yu, Jianxiong Shen, Jinqian Liang

**Affiliations:** 1 Department of Orthopaedic Surgery, Peking Union Medical College Hospital, Peking Union Medical College, Beijing, China

**Keywords:** Wilson’s disase, Thoracic kyphosis, Congential scoliosis

## Abstract

**Background:**

Wilson’s disease (WD) is an autosomal recessive disorder of copper metabolism, which leads to the accumulation of this metal in liver, brain, cornea and kidney. Little is reported about spinal deformity associated with this syndrome. This study is to present a case of thoracic kyphosis occurring in the setting of Wilson’disease and explore the possible association between the two diseases.

**Case presentation:**

Case report and literature review. A previously unreported thoracic kyphosis in Wislon’s disease is decribed. The patient was a 7-year-old Chinese female that underwent a posterior correction, using the Moss-SI spinal system performed at Thoracic 9-Lumbar 1 (T9-L1) levels. At 16-month follow-up, the patient was clinically pain free and well balanced. Plain radiographs showed solid spine fusion with no loss of deformity correction. After evaluating 211 WD patients referred to Peking Union Medical College Hospital from February 1991 to February 2012, the prevalence of congential scoliosis among patients with WD was 5.21% (11/211), much higher than that among general population (1/1000).

**Conclusions:**

To the best of our knowledge, this is the first report of WD with thoracic kyphosis. During sugery, surgeons and anesthesiologists must pay particular attention to the abnormal liver and brain function associated with WD. The prevalence of scoliosis is much higher among patients with WD, indicating a potential association between congential scoliosis and WD. However, the exact mechanism how copper-chelating agents induce scoliosis is unclear.

## Background

Wilson’s disease (WD) is an autosomal recessive disorder of the copper metabolism, leading to the accumulation of this metal in liver, brain, cornea and kidney, with the estimated worldwide prevalence 30 per 1 million [1]. Kinnier Wilson firstly summarized the disease systematically in his thesis on “progressive lenticular degeneration” in 1912 [2]. It is due to a dysfunction of a copper-transporting P-type ATPase that has a crucial role in copper excretion into the bile [3]. WD results from malfunction of ATP7B protein caused by ATP7B gene mutations on the long arm of chromosome 13, inherited as an autosomal recessive trait [4]. Progressive liver cirrhosis, neurologic impairment, and cornea Kayser-Fleischer (K-F) rings and/or renal malfunction are the classical clinical presentations of WD [1]. The therapeutic strategies for WD include low fat diet and penicillamine (PCA).

Since Warnock [5] first observed osteoporosis and spontaneous fractures in a patient suffering from Wilson’s disease, there have been a number of reports describing various skeletal features in this disease, including arthritis and premature osteoporosis [6,7]. However, there are limited reports regarding the association with WD and scoliosis. We here present a case of WD in a 7-year-old girl with unusual presentation: thoracic kyphosis and bilateral femoral head dysplasia.

## Case presentation

**Figure 1:**
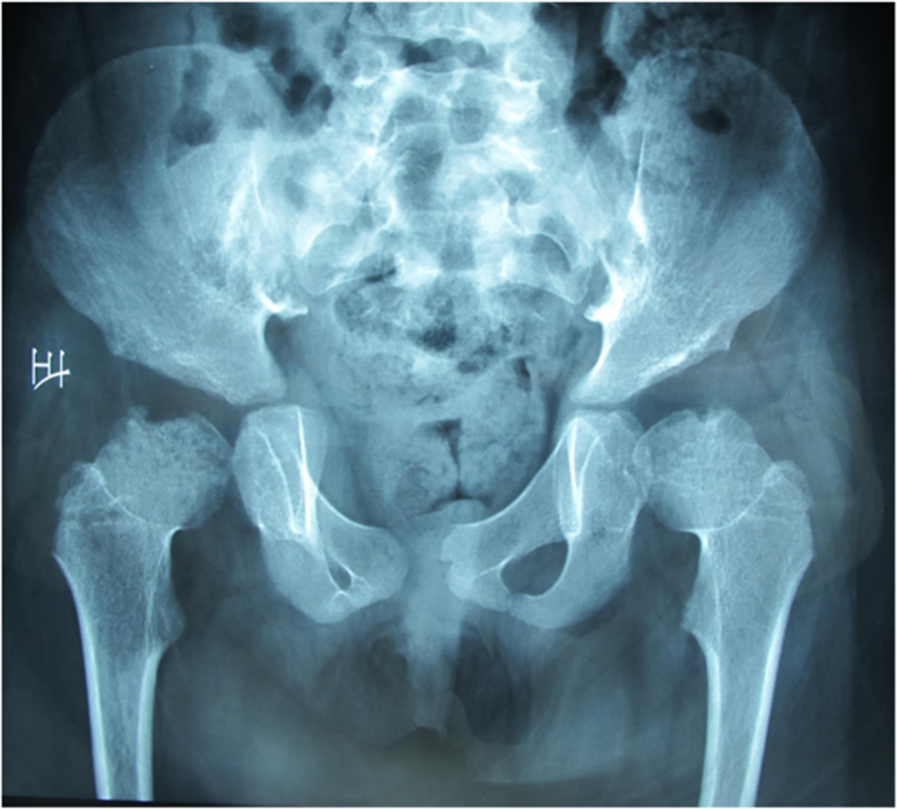
X-rays of the hip revealed bilateral femoral head dysplasia.

**Figure 2:**
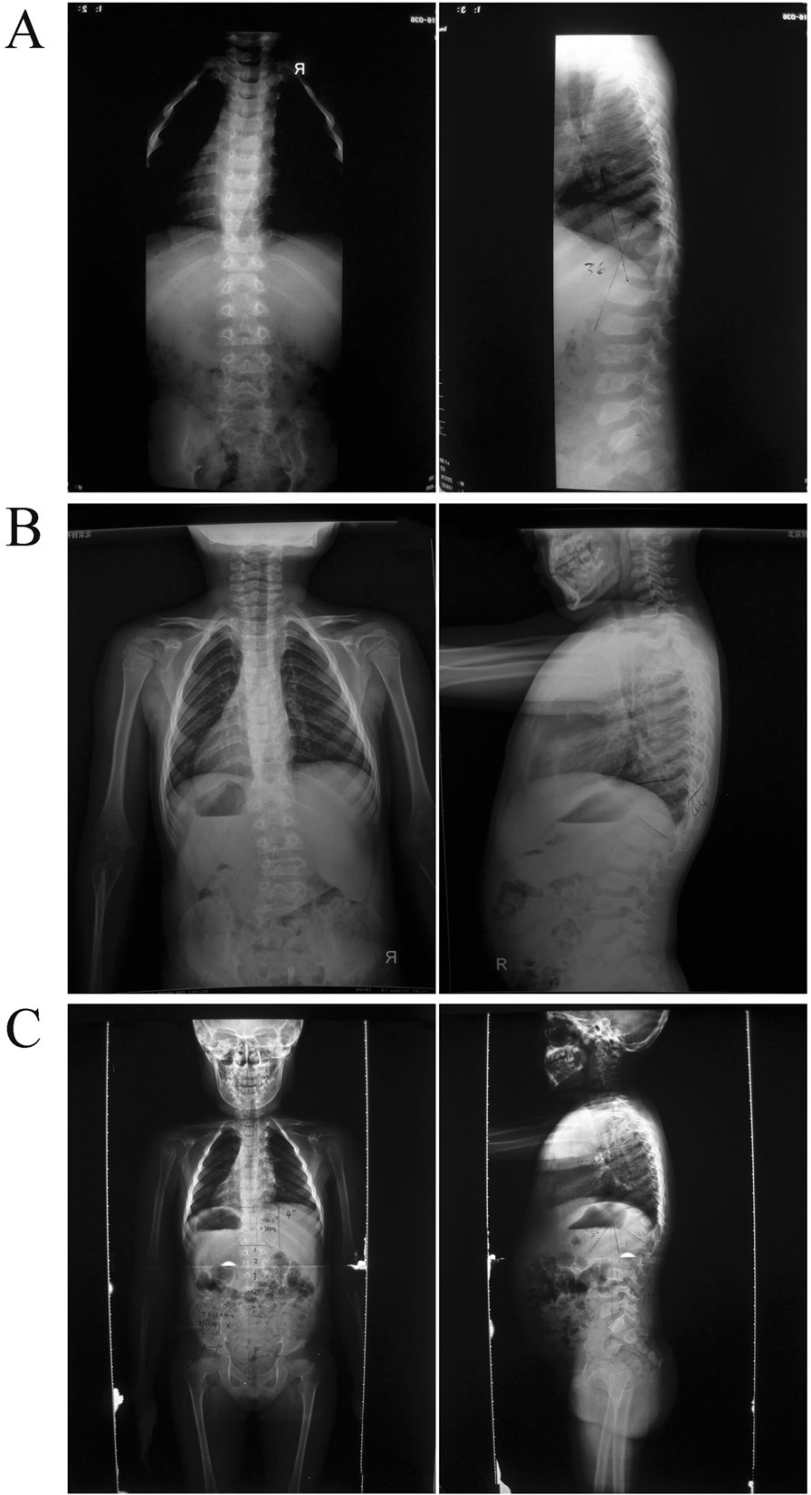
**Preoperative anteroposterior and lateral radiographs. (A)** Preoperative lateral scoliogram showing thoracic 11 vertebral body dysplasia and a thoracic kyphosis curve with cobb of 36. **(B)** After 3 months, the cobb of thoracic kyphosis curve is 44. **(C)** After 6 months, the cobb of thoracic kyphosis curve is 52.

**Figure 3:**
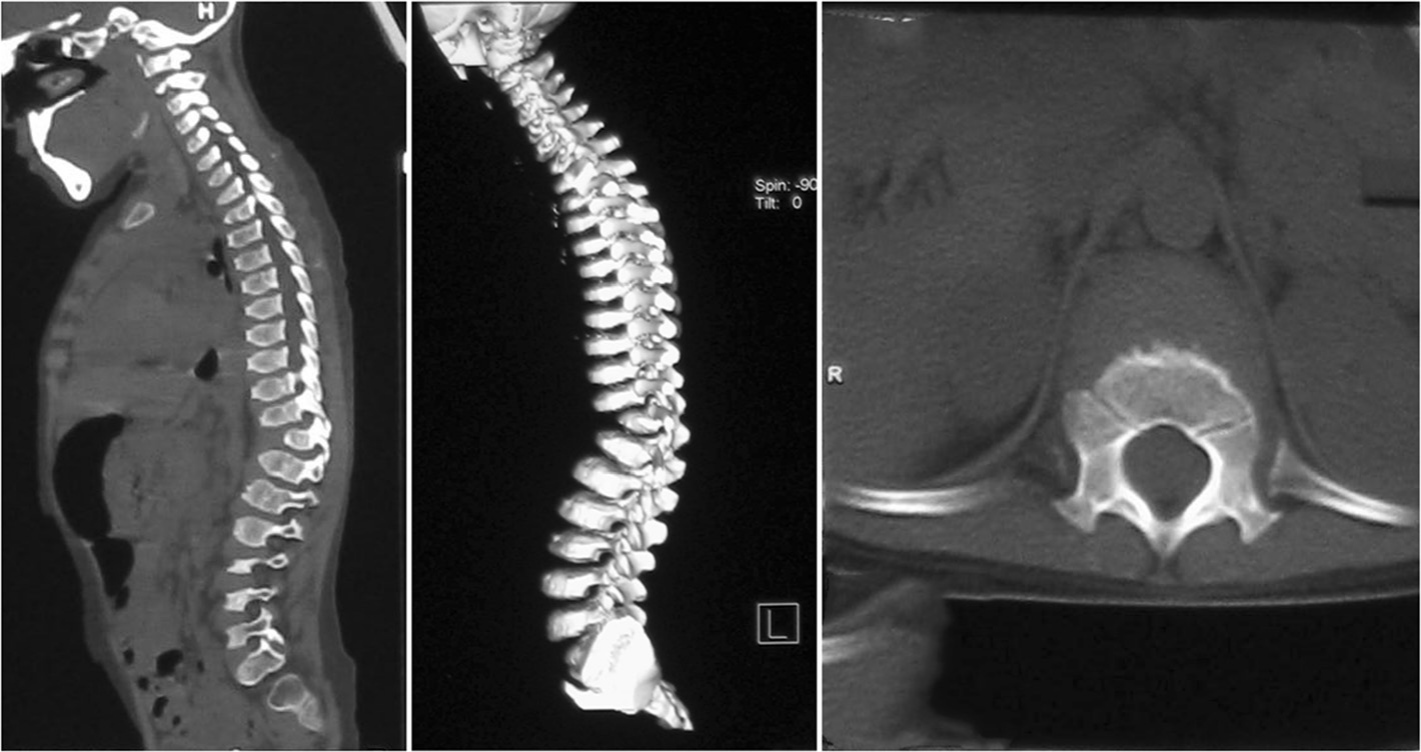
Computed tomography (CT) revealed a thoracic 11 vertebral body dysplasia.

This 7-year-old female first presented to her primary care physician with the complaint of lameness two years ago. X-rays of the hip and the full spine examination revealed bilateral femoral head dysplasia, thoracic 11 vertebral body dysplasia and a thoracic kyphosis curve with Cobb of 36-degree. (Figures 1 and 2), indicating the need of thoracic surgery. Preoperative routine examination of hepatic enzymes discovered a 3-fold increase of aspartate aminotransperas (AST). Antibodies for hepatitis B, S (HBsAg, HbeAg and antibodies to HCV antigens) were normal as well as anti-ANA, anti-AMA, anti-SMA, anti-PCA and anti-LKM. Therefore, she was suggested a delay of surgery and therapeutics of liver function. After half of a year, she was referred to Peking Union Medical College Hospital. Her plain radiographs of the spine showed that the thoracic scoliosis was progressive, with the Cobb increasing from 36-degree to 56-degree (Figure 2), suggesting the surgical correction for her spinal deformity. Magnetic resonance imaging (MRI) revealed no evidence of any spinal cord or canal abnormalities. Computed tomography (CT) revealed a thoracic 11 vertebral body dysplasia (Figure 3). However, the AST and alanine transaminase (ALT) were still high. Given that she had no history of viral hepatitis or alcohol abuse, WD was suspected. So we suggested her to ophthalmology and pediatrics to determine the cause of abnormal liver function. Abnormal liver function, low serum ceruloplasmin, high 24-hour urinary copper excretion and ATP7B mutation were presented. Laboratory examination results were: AST 103 IU/L (5-40 U/L), ALT 68 U/L (5-40 U/L), prothrombin time (INR) 1.1, serum ceruloplasmin 0.03 g/L, 24-hour urinary copper excretion 752 μg, serum copper oxidase absorbance 0.03. Ultrasound of the abdomen revealed hyperechogenic liver parenchyma. Kayser-Fleischer ring on the cornea was not noted. Mutational analysis showed that the proband was homozygote for ATP7B mutation while his parents were heterozygotes and healthy non-carriers. His sister is also a healthy non-carrier. The diagnosis of WD was confirmed combining the clinical presentations and laboratory examinations.

**Figure 4:**
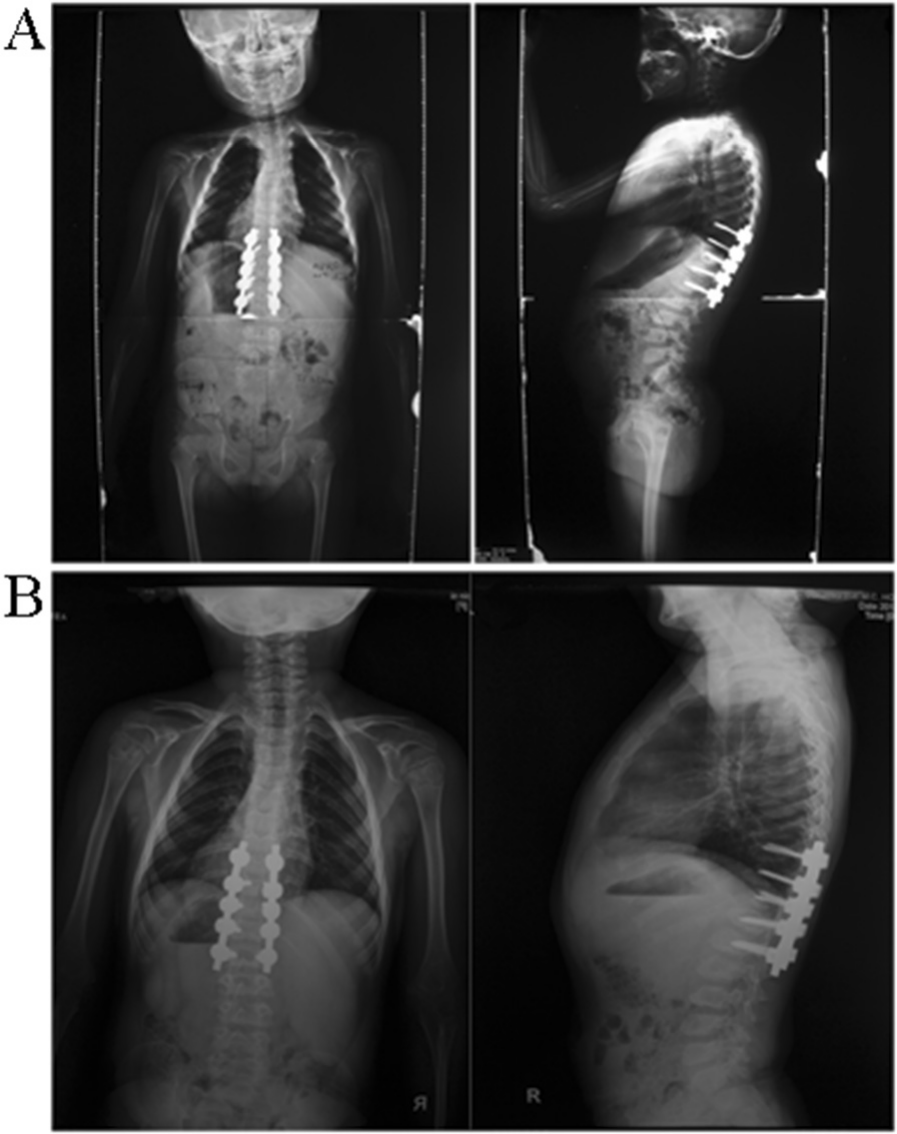
**Postoperative anteroposterior and lateral radiographs. (A)** Postoperative supine anteroposterior view of thoracic spine showing the posterior instrumentation T9 to L1 vertebrae, the cobb of thoracic kyphosis curve is 12. **(B)** Standing anteroposterior and lateral radiographs of 18 months after reoperation.

In February 2012, a posterior correction and fusion at T9-L1 levels was performed, using the Moss-SI spinal system. The total operation time was 2 hours and 5 minutes. Total amount of blood loss was 120 mL. During the operation, the signal of this patient is normal using intraoperative spinal cord monitoring. Postoperatively, there was no sign of liver dysfunction. Postoperative plain X-ray film demonstrated a kyphosis angles correction from 52-degree to 12-degree (correction rate 76.9%) (Figure 4).Eighteen months after thoracolumbar scoliosis correction surgery, the patient went to our cilinics for the follow-up. She was asymptomatic, well balanced in both the sagittal and coronal planes, with solid fusion (Figure 4). Both patient and families were satisfied with the results of surgery. In addition, after adopting the therapy for WD, the liver function is normal.

To study the potential association between congenital scoliosis and WD, we evaluated the spinal deformity of WD patients referred to our hospital from February 1991 to February 2012. The diagnosis of WD was confirmed according to clinical and laboratory examination criteria. A total of 211 WD patients were identified. There were 11 cases of congenital scoliosis in those 211 WD patients. The prevalence of congential scoliosis among patients with WD was 5.21% (11/211), much higher than that among general population (1/1000). The characteristics of patients are described in Table 1.

**Table 1:** Clinicopathologic charateristics of patients with Wilson’s disease

**Parameter**	**Total samples**	**Percentage**	**Average**	**Range**
Age (years)	211		18.1 ± 11.7	3 ~ 59
Gender				
Male	117	55%		
Female	94	45%		
k-F rings				
Present	190	90%		
Absent	21	10%		
Serum copper oxidase absorbance	211		0.051 ± 0.023	0.01 ~ 0.16
scoliosis				
Present	11	5.2%		
Absent	200	94.8%		

Conclusion The prevalence of WD worldwide was estimated to be 30 per 1 million, while it was higher in the Hong Kong Han Chinese populations (1 in 5400) [8]. In addition to the major clinical findings mentioned previously, WD has been reported to be associated with abnormal skeletal manifestations, including arthritis and premature osteoporosis [9]. However, there are limited reports regarding the diagnosis and management of WD and its possible resultant congenital scoliosis. In the present study, we reported the case of a 7-year-old WD case with congential scoliosis. To our konwledge, this is the first report of congential scoliosis in the setting of WD.

WD disease is a genetic disorder with excessive accumulation of copper in the liver and brain due to an inherited defect in its biliary excretion. It is transmitted by autosomal recessive inheritance pattern [10]. Protein ATP7B was proved to be responsible for the transport of copper from the hepatocytes to bile. The gene mutation, located on chromosome 13q1.3, leads to absence or diminished function of ATP7B and a decrease in biliary copper excretion [11,12]. The excessive copper accumulates progressively in the liver, brain, cornea and kidneys and causesg toxic changes in thosse organs. Kayser-Fleischer rings, corneal deposits of copper in Descemet membrane, are present in about 50% of patients with hepatic presentations [1]. In our case, abnormal liver function, low serum ceruloplasmin, high 24-hour urinary copper excretion and ATP7B mutation were presented. However, no evidence of Kayser-Fleischer rings was observed.

Several reportes have revealed the abnormal skeletal manifestations in WD, including osteomalacia, spontaneous fractures, osteoarthritis, osteochondritis dissecans, chondrocalcinosis and subchondral cyst formation. However, congenital scoliosis in WD has not been reported [9,13]. There are no specific guidelines scoliosis operations on patients with WD, but doctors must keep in mind that the progressive liver cirrhosis, neurologic impairment in WD patients could possibly progress to abnormal before surgery. Surgical treatment is the first option for scoliosis patients with WD, accompanied by low fat diet and PCA for WD.

To study the potential association between congential scoliosis and WD, we evaluated the spinal deformity of WD patients referred to our hospital from February 1991 to February 2012. We found that the prevalence of congenial scoliosis among patients with WD was 5.21% (11/211), much higher thanthat among general population (1/1000). However, the exact mechanism how copper-chelating agents induce scoliosis is unclear. Previous studies had shown that the hair copper concentration was significantly higher in patients with scoliosis versus control group [14]. The potential mechanisms in the pathogenesis of congenital scoliosis of WD included genetic variant and excessive accumulation of copper during pregnancy. Further studies were needed to prove the association of gene ATP7B mutation with congential scoliosis in animal model. The main limitations for investigating the association between congenital scoliosis and WD are the sample size, since both of them were rare disease.

## Conclusions

In conclusion, scoliosis is not uncommon among patients with WD. Special laboratory tests, including liver function, serum ceruloplasmin, 24-hour urinary copper excretion, serum carcinoembryonic antigen, and gene ATP7B are needed to confirm the diagnosis. Surgeon and anesthesiologist must pay particular attention to the liver and brain function. There is a potential association between congenital scoliosis and WD. However the exact mechanism how copper-chelating agents induce scoliosis is unclear. Further studies with large patients sample size were needed to confirm the association between the two diseases. Animal models were needed to clarify its mechanisms.

## Consent

Written informed consent was obtained from the patient's parents on behalf of the child for publication of this Case report and any accompanying images. A copy of the written consent is available for review by the Editor of this journal.

## Abbreviations

WD: Wilson’s disease; K-F: Kayser-Fleischer.

## Competing interests

The authors declare that they have no competing interests.

## Authors’ contributions

All authors were involved in the preparation of this manuscript. LZ, XY collected the data and wrote the manuscript. JXS performed the operation and designed the study. JQL summarized the data and revised the manuscript. All authors read and approved the final manuscript.

## Pre-publication history

The pre-publication history for this paper can be accessed here:

http://www.biomedcentral.com/1471-2482/14/71/prepub
